# Pigmented paravenous retinochoroidal atrophy: two case reports and a literature review

**DOI:** 10.5935/0004-2749.2022-0005

**Published:** 2025-08-21

**Authors:** Renato Bezerra Kitahara, Fernando Henrique Flores Teixeira, Aluisio Rosa Gameiro Filho, Carolina Tagliari Estacia, Flavio Mac Cord Medina, Mário Martins dos Santos Motta

**Affiliations:** 1 Hospital Federal dos Servidores do Estado do Rio de Janeiro, Rio de Janeiro, RJ, Brazil

Dear Editor,

Pigmented paravenous retinochoroidal atrophy (PPRCA) is a rare, commonly asymptomatic, bilateral, symmetrical, and non-or slowly-progressive retinal degeneration^(1-5)^.

The characteristic changes in the posterior pole are pigment accumulation in the bone spicule pattern and the areas of retinochoroidal atrophy that follow the course of the retinal veins^(1-5)^. Multimodal analysis fa cilitates the diagnosis and comprehension of this pathology and is performed using fundus autofluorescence (FAF), fundus fluorescein angiography, optical coherence tomography (OCT), electroretinogram (ERG), and computerized visual campimetry.

This paper presents two classic cases of PPRCA from the same ophthalmology hospital, both with important clinical characteristics and changes in the complementary examinations.

## Case 1

Was a 21-year-old black woman, born and residing in Rio de Janeiro, with no complaints, who visited the general ophthalmology outpatient clinic of the Hospital Federal dos Servidores do Estado for routine consultation. She had no comorbidities and no history of eye trauma or inflammatory and infectious eye disease. Her family history of the ocular disease was also negative.

On ophthalmological examination, the corrected visual acuity was found to be 20/20 in both eyes. The anterior segment and anterior vitreous did not present with any alteration.

**Figure 1:**
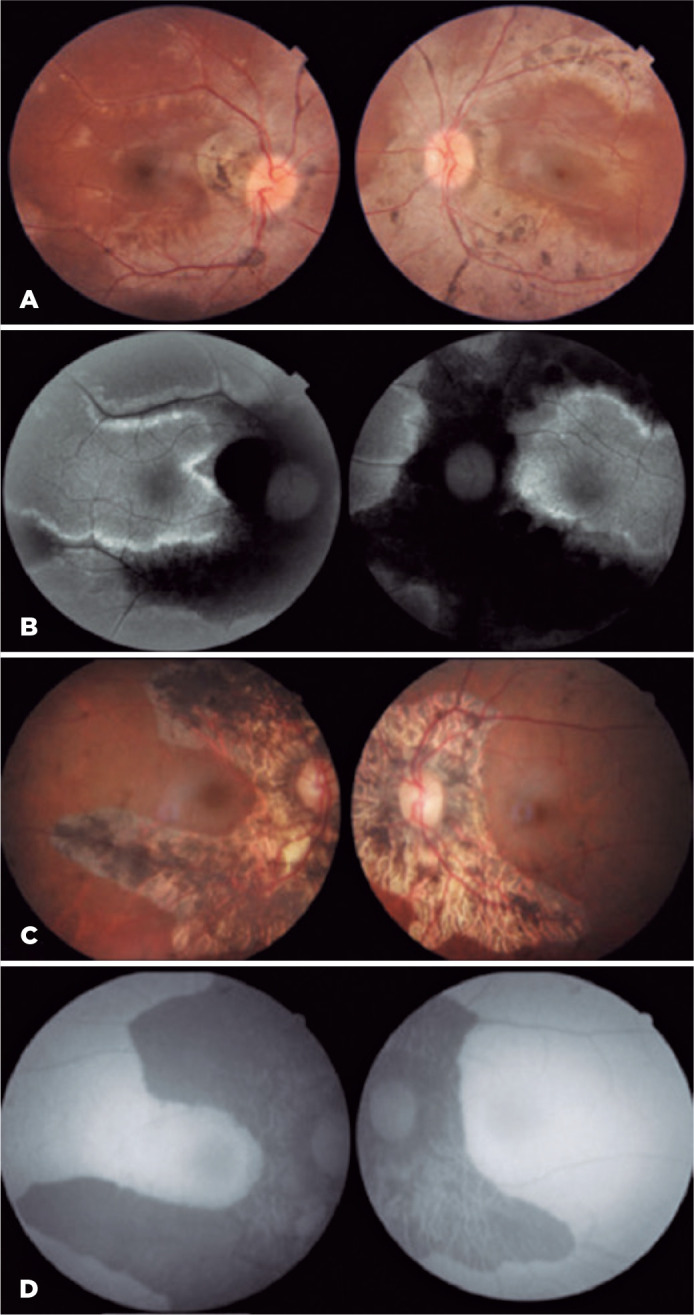
(A) Retinography: Areas of retinochoroidal atrophy and pigment mobilization in the bone spicule pattern involving the optic disc, following the course of the retinal veins and extending to the retinal equator. (B) Autofluorescence: Hypoautofluorescence along the retinal veins and hyperautofluorescence lines outlining the hypoautofluorescent areas. (C) Retinography: Retinal pigmented epithelium (RPE) atrophy and pigmentary clumps around the optic disc and following the pathway of blood vessels, sparing the maculas. (D) Autofluorescence: Shows hypoautofluorescence along with the paravenous areas and no areas of hyperautofluorescence.

Funduscopic examination revealed areas of retinochoroidal atrophy and pigment mobilization in the bone spicule pattern involving the optic disc, following the course of the retinal veins and extending to the retinal equator (Figure 1A).

Autofluorescence revealed hypoautofluorescence along the retinal veins and hyperautofluorescence lines outlining the hypoautofluorescent areas (Figure 1B).

**Figure 2:**
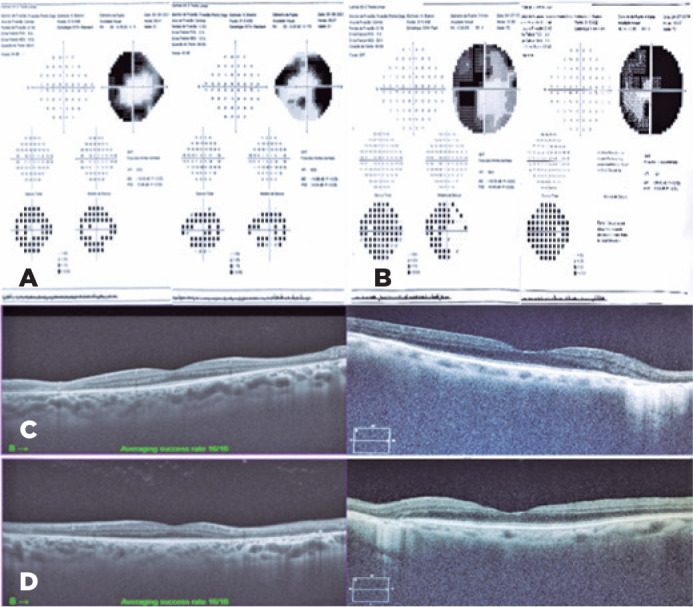
(A) 24-2 Humphrey visual field: The presence of deep arcuate scotoma in the right eye and a ring-shaped scotoma in the left eye. (B) 30-2 Humphrey visual field: A ring-shaped scotoma in both eyes. (C) Optical coherence tomography (OCT): Preserved foveal profile, average macular thickness, and intact retinal layers. In the areas of the lesions, there was a loss of the outer retinal layers in both eyes. (D) OCT: Loss of the outer retinal layers along with a reverse shadowing sign due to RPE atrophy in the areas of the lesions in both eyes.

OCT of the maculas revealed preserved foveal profile, average macular thickness, and intact retinal layers. In the areas of the lesions, there was a loss of the outer retinal layers (Figure 2C).

A computerized visual field test revealed the presence of deep arcuate scotoma in the right eye and ring-shaped scotoma in the left eye, which corresponded to the areas of atrophy and pigment accumulation (Figure 2A).

Ophthalmological examination of the family members did not reveal any pathological changes.

## Case 2

Is a 71-year-old woman with complaints of progressive visual loss in both eyes. She was previously diagnosed with a bilateral cataract. She had diabetes and systemic hypertension, both well controlled with medication.

Her best-corrected visual acuity was 20/40 in both eyes and her potential acuity meter was 20/20 in both eyes. Slit-lamp examination revealed a symmetric cataract (nuclear 2+/4+) and no other abnormalities in the anterior segment. Fundoscopy disclosed retinal pigmented epithelium (RPE) atrophy and pigmentary clumps around the optic disc and following the pathway of blood vessels, sparing the maculas (Figure 1C). FAF showed geographic hypoautofluorescence along with the paravenous areas and no areas of hyperautofluorescence (Figure 1D). OCT showed the loss of the outer retinal layers in the areas of the lesions (Figure 2D) and a computerized visual field test revealed a ring-shaped scotoma in both eyes (Figure 2B). Systemic screening for infectious or rheumatic diseases was negative, with no family history of ocular diseases.

PPRCA is a rare retinal degeneration that presents with pigment accumulations in the posterior pole in a bone spicule pattern and the areas of retinochoroidal atrophy that follow the course of the retinal veins^(1-5)^. Its etiology remains unknown, and it is unclear whether there is a genetic, inflammatory, or infectious association and whether it is systemic or ocular^(1-3,5)^. No genetic study was performed on our patients considering no family history in both the cases.

As for the present cases, patients are usually diagnosed during routine consultation based on typical appearances on fundoscopy. Changes in the complimentary examination may help confirm the diagnosis^(1,2)^.

In areas of chorioretinal atrophy, autofluorescence revealed hypoautofluorescent areas surrounded by hyperautofluorescent lines^(2,3)^, as noted in the first case.

Fluorescein angiography reveals areas of hyperfluorescence due to window-defect in the areas of RPE atrophy and blockage of hypofluorescence in the areas of pigment accumulation^(2)^. No fluorescein leakage was observed at any stage of examination^(2)^.

The differential diagnoses encompass inflammatory diseases and degenerations along the course of the retinal veins with retinochoroidal atrophy^(2,4)^. These include retinitis pigmentosa, helicoid peripapillary chorioretinal degeneration, serpiginous choroiditis, angioid streaks, sarcoidosis, syphilis, tuberculous choroiditis, toxoplasmosis, among others^(2)^. The present cases did not present any features compatible with those of infectious or inflammatory pathology.

Although there is no specific treatment for PPRCA^(2,4)^, it showed a good prognosis because it does not progress at all or progresses slowly^(1-5)^.

Both patients were reassessed every 6 months and showed no visual changes, with no progression of the lesion to the posterior pole.
